# HOTAIR as a diagnostic and prognostic biomarker of gastrointestinal cancers: an updated meta-analysis and bioinformatics analysis based on TCGA data

**DOI:** 10.1042/BSR20222174

**Published:** 2023-03-29

**Authors:** Jinyou Yang, Shuyu Xu, Shaozhuo Wang, Xuyan Zou, Mingxiu Duan, Qiaoling Zhang, Chao Wang

**Affiliations:** 1 Department of Health and Rehabilitation, Jiangsu College of Nursing, Huai’an, Jiangsu 223005, P. R. China; 2 Key Lab of Modern Toxicology of Ministry of Education, Center for Global Health, Department of Occupational Medicine and Environmental Health, School of Public Health, Nanjing Medical University, 101 Longmian Avenue, Nanjing 211166, P. R. China; 3 Department of Nursing and Midwifery, Jiangsu College of Nursing, Huai’an, Jiangsu 223005, P. R. China

**Keywords:** cancer, digestive system, Hox transcript antisense intergenic RNA, long noncoding RNA, meta-analysis, prognosis

## Abstract

Gastrointestinal cancers are the most common type of cancer affecting humans. High expression of HOX transcript antisense intergenic RNA (HOTAIR), a long noncoding RNA (lncRNA), in various types of different tumors may be associated with poor prognosis. In the present study, we performed a meta-analysis of the relationship between HOTAIR expression and gastrointestinal cancers. Five databases were comprehensively searched for all literature until January 2023. Moreover, the target genes of HOTAIR were predicted by coexpression analysis based on The Cancer Genome Atlas (TCGA) gene expression matrix for six gastrointestinal cancer types. Finally, the mechanism through which HOTAIR affects tumors of the digestive system was systematically reviewed. Our results showed that the high HOTAIR expression group had worse outcomes with a pooled hazard ratio (HR) of 1.56 (95% confidence interval [CI] = 1.38–1.75, *P*<0.001). Furthermore, HOTAIR was identified as an unfavorable prognostic factor for overall survival (OS) in the esophageal carcinoma (ESCA) and gastric cancer (GC), as the HR were 1.94 and 1.58, respectively. The high correlation between the expression of homeobox C (HOXC) family genes and HOTAIR, with correlation coefficients of 0.863 (*HOXC11*), 0.664 (*HOXC10*), 0.645 (*HOXC8*), and 0.581 (*HOXC12*). The ‘*cell cycle’* pathway and pathways relating to infections, namely *‘herpes simplex virus 1 infection’ and ‘complement and coagulation cascades’* were significantly enriched in Kyoto Encyclopedia of Genes and Genomes (KEGG) analysis. Also, we perform a systematic review to summarize the related oncogenic mechanism of HOTAIR. In conclusion, the HOTAIR has been identified as a potential prognostic factor in patients with gastrointestinal cancers.

## Introduction

Globally, gastrointestinal cancers are a serious form of cancer and a threat to human health. For example, gastric cancer (GC) is the fifth most common cancer and the third leading cause of cancer-related deaths, pancreatic cancer is the seventh most common cause of cancer death, and liver cancer is the fourth most common cause of cancer-related deaths [1–3]. With the development of modern medicine, the diagnostic techniques and treatment methods for gastrointestinal tumors have improved dramatically, the 5-year survival rate remains low, and several types of gastrointestinal cancers still lack effective screening methods, such as pancreatic cancer [4]. Many studies have developed different models to find effective tumor biomarkers for early diagnosis [5,6], but the predictive ability and function of those biomarkers need further study and verification.

Several genetic mutations and epigenetic changes are involved in tumor initiation and progression. Epigenetic changes, including long noncoding RNAs (lncRNAs), have emerged as one of the most powerful biomarkers, as genomic and epigenomic changes are implicated in nearly all gastrointestinal cancers [7,8]. LncRNAs refer to noncoding RNA with more than 200 nucleotides and play an important role in many cellular processes, including the cell cycle, differentiation, and metabolism [9]. LncRNAs regulate gene expressions at epigenetic, transcriptional, and post-transcriptional levels [10], many lncRNAs are now treated as important cancer-related factors. This may be due to their ability to interact with DNA, RNA, or proteins and participate in alternative splicing [11]. According to its relative position with coding genes, lncRNA can be divided into five types, including sense lncRNA, antisense lncRNA, bidirectional lncRNA, intronic lncRNA, and larger intergenic noncoding RNA [12]. In cancer, lncRNAs have been shown to affect tumor energy metabolism [13], epithelial to mesenchymal transition (EMT) [14], and reprogramming of tumor metastasis, proliferation, and migration [15,16]. Moreover, accumulating evidence suggests that lncRNAs could be serving as decoys, scaffolds, competing for endogenous RNAs (ceRNAs), etc. [17]. It is noteworthy that the latest research demonstrated that lncRNAs regulate biological processes by encoding short peptides [18], especially in hepatocellular carcinoma, pancreatic ductal adenocarcinoma, etc. [19]. Therefore, as the important roles of lncRNA, researchers have started to pay more attention to the function of lncRNA, and it is being investigated as a predictor and drug target for early gastrointestinal cancers.

Our previous article demonstrated that lncRNA MALAT1 is associated with the progression of digestive malignant tumors, which suggests that it may serve as a prognostic factor for digestive system malignancies [20]. In addition to MALAT1, lncRNA H19, HOX transcript antisense intergenic RNA (HOTAIR), PRNCR1, HOTTIP, and CCAT1 have also been widely reported to be involved in human tumorigenesis and metastasis [21]. HOTAIR is a 2.2‐kb-long noncoding RNA located on chromosome 12q13.13, transcribed from the homeobox C gene (*HOXC*) locus, and has been recognized as a regulator of cancer [22,23]. HOTAIR is a well-known lncRNA that was first characterized in breast cancer [24]. As the research in this area has progressed, numerous HOTAIR-related studies have been published, and aberrant expression of HOTAIR has been associated with cancers at various sites, including lung cancer [25], cervical cancer [26], breast cancer [27], glioblastoma [28], and gastrointestinal cancers [29–31]. In lung cancer, HOTAIR is an obvious indicator of cell cycle dysregulation, which can affect the EMT and β-catenin signaling pathways by inhibiting the cell cycle from G1 to S phase, and promoting the proliferation, metastasis, and invasion of NSCLC cell lines [32]. In cervical cancer, HOTAIR promotes cell proliferation, migration, invasion, and autophagy, inhibits apoptosis, stimulates angiogenesis, accelerates cell cycle progression, and induces EMT [26]. In breast cancer, HOTAIR is critical to tumor progression and can directly or indirectly regulate key cellular processes such as cell proliferation, cell invasion, EMT, self-renewal, metastasis, and drug resistance. The main function of HOTAIR in glioblastoma is to promote cell proliferation and migration, and inhibit cell apoptosis [28]. These facts suggest that HOTAIR is an excellent therapeutic target for broad-spectrum cancer treatment.

Furthermore, high expression of HOTAIR is associated with drug resistance and poor overall survival (OS) in many cancers [33]. Numerous meta-analyses have focused on the expression of HOTAIR and its value in tumor prognosis and diagnosis, such as digestive system carcinomas [34], hepatocellular carcinoma [35], colorectal cancer (CRC) [36], and head and neck tumors [37]. Meanwhile, studies focusing on the gene polymorphism of HOTAIR and the value of tumor prognosis and diagnosis [38–40], such as lung cancer [41], have also been published. However, these studies are over 3-year old, and a large amount of research has been published since then. For example, HOTAIR has recently been shown to play an important role in gastrointestinal cancers, correlating with metastasis, proliferation, migration, EMT, cell cycle, and so on [42]. HOXC11 positively regulates the lncRNA HOTAIR and is associated with poor prognosis in colon adenocarcinoma (COAD) [43]. In esophageal squamous cell carcinoma, down-regulation of HOTAIR inhibits invasion and migration via up-regulation of miRNA-204 [44]. Furthermore, HOTAIR can target miRNA, and activate related pathways, thus promoting GC proliferation, metastasis, and EMT [45]. Overall, HOTAIR plays an important role in gastrointestinal cancers and could potentially be used as a biomarker. It is also necessary to perform an updated meta-analysis and a literature review of the molecular mechanisms for the role of HOTAIR in GI cancers.

The present study further confirmed the role and prognostic ability of HOTAIR in gastrointestinal tumors, demonstrated the prognostic value of HOTAIR in gastrointestinal tumors by meta-analysis, and showed the correlation between HOTAIR and protein-coding genes. Through a systematic review, we predicted and summarized the HOTAIR expression network and, ultimately, the important molecular mechanisms in tumorigenesis and progression.

## Materials and methods

### Literature search strategy

The databases PubMed (https://www.ncbi.nlm.nih.gov/pubmed), Embase (https://www.embase.com/), Web of Science (http://apps.webofknowledge.com/), the Scopus database (http://www.elsevier.com/online-tools/scopus), and the China National Knowledge Infrastructure (http://www.cnki.net/) were searched from their inception up to January 2023 for studies to include in our meta-analysis. The following key terms were searched under the principles of ‘patient/population, intervention, comparison and outcomes, PICO’: ‘long noncoding RNA’, ‘lncRNA’, ‘HOTAIR’, ‘HOX transcript antisense RNA’, ‘neoplasm’, ‘cancer’, ‘tumor’, ‘colorectal cancer’, ‘rectal cancer’, ‘esophageal carcinoma’, ‘pancreatic cancer’, ‘hepatocellular carcinoma’, ‘gastric cancer’, ‘cholangiocarcinoma’, ‘survival’, ‘follow-up’, ‘outcome’, and ‘predictor’. The bibliographies of the retrieved articles, relevant reviews, and conference reports were also checked for relevant studies. More specific retrieval strategies were provided in Supplementary Table S1.

### Inclusion and exclusion criteria

Eligible studies had to meet the following criteria:

- diagnosis of digestive system cancer must be confirmed: pathological examination and diagnosis were performed by an independent senior oncologist according to official pathological diagnostic criteria;
- the expression level of HOTAIR was examined in control and tumor tissues;
- the description of expression measurement of HOTAIR must be clear;
- the cutoff value for HOTAIR must be described;
- the relationship between the level of HOTAIR and the patient survival time was analyzed; and
- the hazard ratios (HRs) and 95% confidence intervals (CIs) for the survival rate were reported or could be calculated.

The exclusion criteria were as follows:

- studies without usable data;
- animal studies, case reports, letters, reviews, and conference abstracts;
- meta-analysis studies;
- studies that included patients with benign tumors;
- studies that included patients after drug treatment; and
- studies that did not contain or could not calculate HRs, 95% CIs, *P*-values, and survival data.

### Data extraction and quality assessment

Two individuals of the authors extracted data from each study and independently estimated. The extracted data included the first author, year of publication, country, cancer type, sample size, tumor-node-metastasis stage (TNM), follow-up months, adjuvant therapy before surgery (yes or no), cutoff value, method of HOTAIR expression-level measurement, survival rates [OS and disease-free survival (DFS)], HRs, and 95% CIs. In cases where the HRs and 95% CIs were not directly reported, Engauge Digitizer software version 4.1 (http://digitizer.sourceforge.net/) was used to plot the Kaplan–Meier curves and extract the multiple survival rates to estimate the HRs and 95% CIs [46]. Quality assessment was performed using the Newcastle–Ottawa quality assessment scale (NOS). NOS criteria scores range from 0 (lowest) to 9 (highest), and a NOS score ≥6 is considered a high-quality study [47].

### Bioinformatics analysis

Based on the gene expression matrix of six gastrointestinal tumors [liver hepatocellular carcinoma (LIHC), stomach adenocarcinoma (STAD), esophageal carcinoma (ESCA), pancreatic adenocarcinoma (PAAD), COAD, and cholangiocarcinoma (CHOL)] download from The Cancer Genome Atlas (TCGA, as mentioned in our previous study [48,49]. The mRNA expression profiles and the corresponding clinical data of 312 GI cancer patients of Asian ancestry were obtained. Raw mRNA expression data were normalized by [log2 (data+1)] for further statistical analysis and the ‘edgeR’ package was used to identify differentially expressed lncRNAs and protein-coding genes (PCGs) [50]. Then, the lncRNA-mRNA coexpression network was constructed to predict the potential biological functions of lncRNA [51]. We examined the correlation between the expression level of lncRNA HOTAIR and each PCG using two-sided Pearson correlation coefficients and the z-test. The PCGs positively or negatively correlated with the lncRNA HOTAIR were considered lncRNA HOTAIR-related PCGs (|Pearson correlation coefficient| > 0.5 and *P*<0.001). Kyoto Encyclopedia of Genes and Genomes (KEGG) analysis was used for functional enrichment analysis, which was conducted by the ‘stringi’ package, ‘biocmanager’ package, ‘pathview’ package, and ‘clusterprofiler’ package. All those pathways were mapped based on the KEGG database and searched for significantly enriched KEGG pathways at *P*<0.05 level. R software (R version 4.2.1) was used for statistical analysis and to plot the data.

### Statistical analysis

HRs and 95% CIs were extracted to investigate the association between the expression level of HOTAIR and the survival rates of patients with malignant digestive system tumors. An HR value not equal to 1 was considered statistically significant, while an HR value greater than 1 indicated a worse survival outcome in the high HOTAIR expression group. The random and fixed effects models were compared and used to estimate the pooled HR. The Q-test was performed, and the *I*^2^ statistic was determined to quantify the heterogeneity across the studies. An *I*^2^ statistic of more than 50% and a Q-test result with *P*<0.05 indicated strong heterogeneity, and studies were grouped to reduce heterogeneity further. In addition, a sensitivity analysis was performed. Then, we investigated publication bias using funnel plots, Egger’s test, and Begg’s test [52]. All meta-analyses were performed using Stata 15.0 software (Stata Corp. College Station, TX, U.S.A.). *P*<0.05 was considered to indicate that the results were statistically significant.

## Results

### Literature search and study characteristics

**Figure 1:**
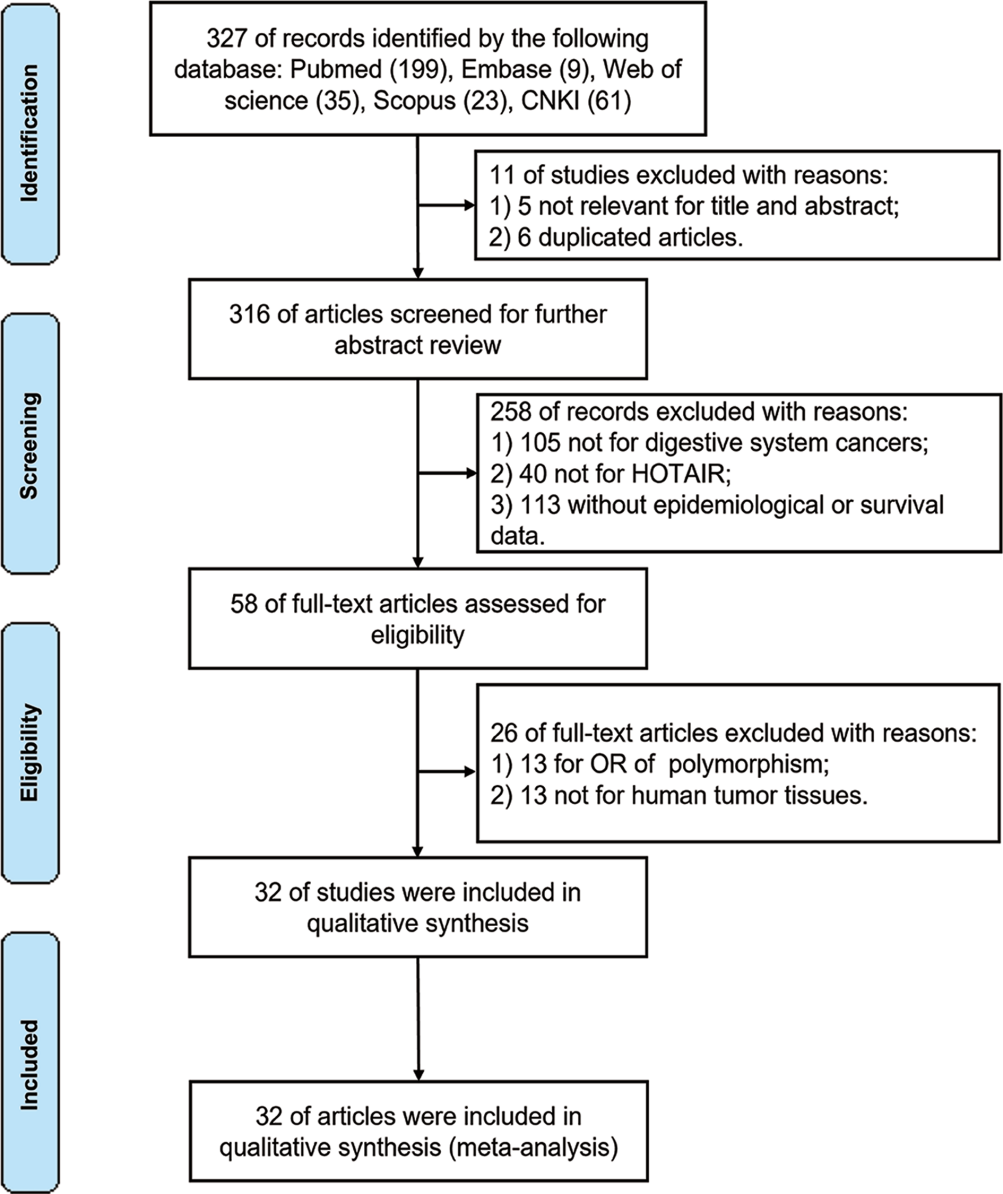
Search strategy and study selection Abbreviations: CNKI, China National Knowledge Infrastructure; OR, odds ratio.

A total of 304 articles were retrieved according to the criteria. After careful analysis, 32 studies from 2011 to 2023 were included in the meta-analysis (Figure 1). Among them, six were related to ESCA [53–58], 14 were for GC [59–72], seven were CRC [29,73–78], three were hepatocellular carcinoma [30,79,80], one was pancreatic cancer [81], and one was CHOL [82]. Survival data for 2901 patients were included in the meta-analysis, with a median sample size of 91 patients (range 33–168). Of the 32 studies, 24 were performed in China, four in Japan, one in Korea, one in Lithuania, one in Denmark, and one in Czech. The main characteristics of these studies are summarized in Table 1. All patients were divided into either high or low HOTAIR expression groups according to the HOTAIR cutoff value. A total of 29 studies reported the OS rate, while four studies reported the DFS rate. HRs and 95% CIs were extracted directly from 17 studies and were extracted and calculated from 15 studies using a survival curve with the help of Engauge Digitizer. The Newcastle–Ottawa Quality Rating Scale was used to assess the quality of the 32 studies (Supplementary Table S2).

**Table 1:** Main characteristics of all studies included in the meta-analysis

First author	Country	Cancer type	Sample size	TNM stage	Follow-up time, mouth	AT (yes/no)	Quantification of expression	Cutoff value	Multivariate analysis	HR (95% CI)	NOS
Geng, 2011 [30]	China	HCC	50	NA	NA	50/0	qRT-PCR	ROC cutoff	No	DFS: 2.21 (1.08, 4.50)	7
Yang, 2011 [80]	China	HCC	60	NA	18.6^c^	NA	qRT-PCR	Median expression	No	DFS: 3.56 (1.67, 7.63)	7
Kogo, 2011 [29]	Japan	CRC	100	NA	36^c^	100/0	qRT-PCR	H/G^d^ = 0.27	Yes	OS: 3.18 (0.71, 14.33)	7
Endo, 2013 [59]	Japan	GC^a^	36	I–IV	>60	36/0	qRT-PCR	Median expression	No	OS: 0.97 (0.11, 8.66)	7
Endo, 2013 [59]	Japan	GC^b^	32	I–IV	>60	32/0	qRT-PCR	Median expression	No	OS: 1.80 (0.21, 15.21)	7
Xu, 2013 [69]	China	GC	83	I–IV	>72	83/34	qRT-PCR	Median expression	Yes	OS: 1.30 (0.59, 2.87)	7
Ishibashi, 2013 [79]	Japan	LIHC	64	NA	NA	NA	qRT-PCR	Median expression	No	OS: 3.26 (1.3, 7.9)	6
Chen, 2013 [53]	China	ESCA	78	NA	38^c^	78/0	qRT-PCR	Median expression	Yes	OS: 2.40 (1.35, 4.28)	7
Ge, 2013 [54]	China	ESCA	137	I–IV	NA	137/0	qRT-PCR	ROC cutoff	Yes	OS: 3.16 (1.53, 6.52)	7
Lv, 2013 [57]	China	ESCA	93	I–IV	>60	93/0	qRT-PCR	Median expression	Yes	OS: 1.66 (1.01, 2.72)	6
Li, 2013 [58]	China	ESCA	156	I–IV	>60	156/0	qRT-PCR	Median expression	Yes	OS: 1.91 (1.1, 3.4)	5
Guo, 2014 [60]	China	GC	102	I–IV	>60	102/0	qRT-PCR	Median expression	Yes	OS: 1.77 (1.06, 2.95)	8
Okugawa, 2014 [61]	Japan	GC	150	I–IV	26^c^	NA	qRT-PCR	Median expression	Yes	OS: 2.09 (1.26, 3.45)	8
Lee, 2014 [65]	Korea	GC	50	I–III	>48	NA	qRT-PCR	Median expression	No	DSF: 2.21 (0.53, 9.16)	7
Liu, 2014 [66]	China	GC	78	II–IV	>42.5	78/0	qRT-PCR	Median expression	No	OS: 3.63 (2.06, 6.39)	7
Wu, 2014 [75]	China	CRC	120	I–IV	55.5^c^	120/0	qRT-PCR	T/N^e^ > 5	Yes	OS: 3.92 (1.23, 12.50)	7
										DFS: 3.88 (1.37, 10.98)	
Zhao, 2015 [72]	China	GC	168	III–IV	>60	168/0	qRT-PCR	Median expression	Yes	OS: 1.47 (1.04, 2.06)	7
Ye, 2016 [71]	China	GC	98	I–III	NA	98/0	qRT-PCR	Median expression	Yes	OS: 2.68 (1.41, 5.07)	7
Luo, 2016 [74]	China	CRC	80	I–IV	>60	80/0	qRT-PCR	Median expression	No	OS: 2.03 (1.14, 3.59)	7
Xu, 2017 [55]	China	ESCA	40	I–IV	>35	NA	qRT-PCR	Median expression	No	OS: 2.45 (0.94, 6.39)	6
Jab, 2017 [56]	Denmark	ESCA	40	I–IV	22^c^	40/20	qRT-PCR	The two-thirds expression	Yes	OS: 3.40 (1.20, 9.60)	7
Xiao, 2018 [77]	China	CRC	104	I–IV	NA	104/0	qRT-PCR	Median expression	No	OS: 1.93 (0.90, 4.13)	6
Qin, 2018 [82]	China	CHOL	33	I–IV	NA	33/0	qRT-PCR	Median expression	Yes	OS: 1.89 (1.03, 3.46)	7
Xun, 2019 [62]	China	GC	70	NA	NA	NA	qRT-PCR	Median expression	No	OS: 1.34 (0.38, 4.65)	7
Dong, 2019 [63]	China	GC	40	I–IV	>60	32/0	qRT-PCR	Median expression	No	OS: 1.35 (0.42, 4.34)	7
Jia, 2019 [64]	China	GC	317	I–IV	NA	317/0	qRT-PCR	Median expression	Yes	OS: 1.48 (1.27, 1.88)	7
Xu, 2019 [70]	China	GC	54	I–IV	>48	54/0	qRT-PCR	Median expression	No	OS: 2.68 (1.37, 5.25)	6
Ma, 2019 [81]	China	PAAD	78	I–IV	>60	NA	qRT-PCR	Median expression	No	OS: 1.06 (0.62, 1.83)	7
Zhang, 2020 [67]	China	GC	87	I–IV	>35	NA	qRT-PCR	Median expression	No	OS: 2.02 (0.86, 4.75)	6
Liu, 2020 [73]	China	CRC	71	NA	>60	NA	qRT-PCR	Median expression	No	OS: 2.60 (0.99, 6.79)	7
Jia, 2020 [76]	China	CRC	32	II–IV	>32	32/32	qRT-PCR	H/G^d^ = 1.967	Yes	OS: 1.21 (1.04, 2.13)	7
Petkevicius, 2022 [68]	Lithuania	GC	127	I–IV	83	81/0	qRT-PCR	Median expression	Yes	OS: 3.13 (1.12, 8.75)	6
Svoboda, 2014 [78]	Czech	CRC	73	I–IV	54	0/73	qRT-PCR	Median expression	Yes	OS: 4.43 (1.02, 19.19)	8

Abbreviations: AT, adjuvant therapy prior to surgery; CHOL, Cholangiocarcinoma; CRC, colorectal cancer; DFS, disease-free survival; ESCA, esophageal carcinoma; GC, gastric cancer; HCC, hepatocellular carcinoma; NA, not available; NOS, Newcastle–Ottawa quality assessment scale; OS, overall survival; PAAD, pancreatic adenocarcinoma; PFS, progression-free survival.

a Intestinal type.

b Diffuse type.

c Data are presented as the median.

d H/G, HOTAIR expression/GAPDH expression.

e T/N, HOTAIR in tumor/in normal.

### The prognostic value of HOTAIR in digestive system malignancies

**Figure 2:**
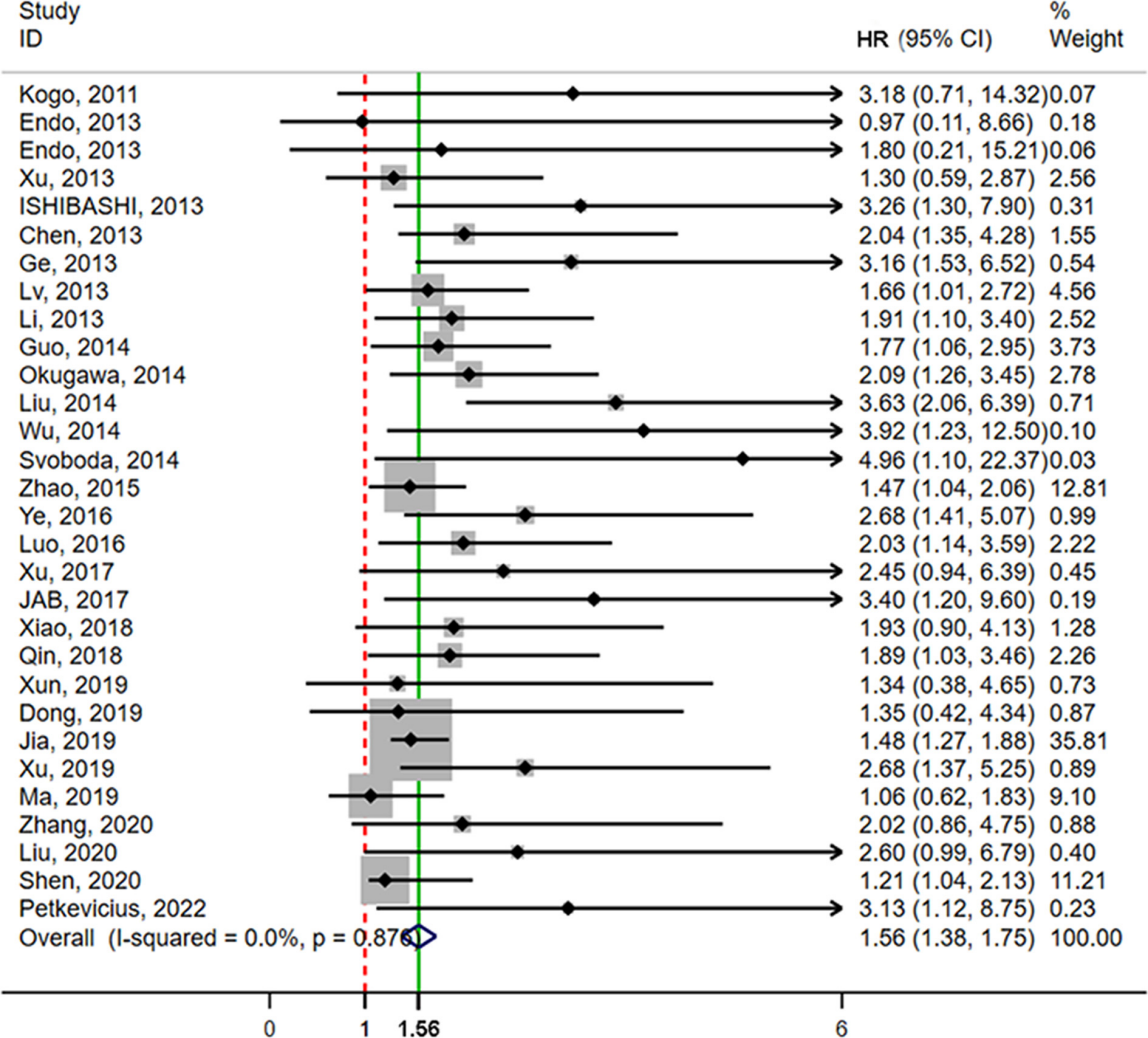
Forest plot for the analysis of the association between HOTAIR expression and OS of digestive system malignancies The point estimate was bounded by a 95% CI. The solid green line represents the OS summary estimate, in which the 95% CI is shown by its width. The dotted vertical red line describes the null value (HR = 1), which represents no increased risk for the outcome. Abbreviations: CI, confidence interval; HR, hazard ratio.

**Figure 3:**
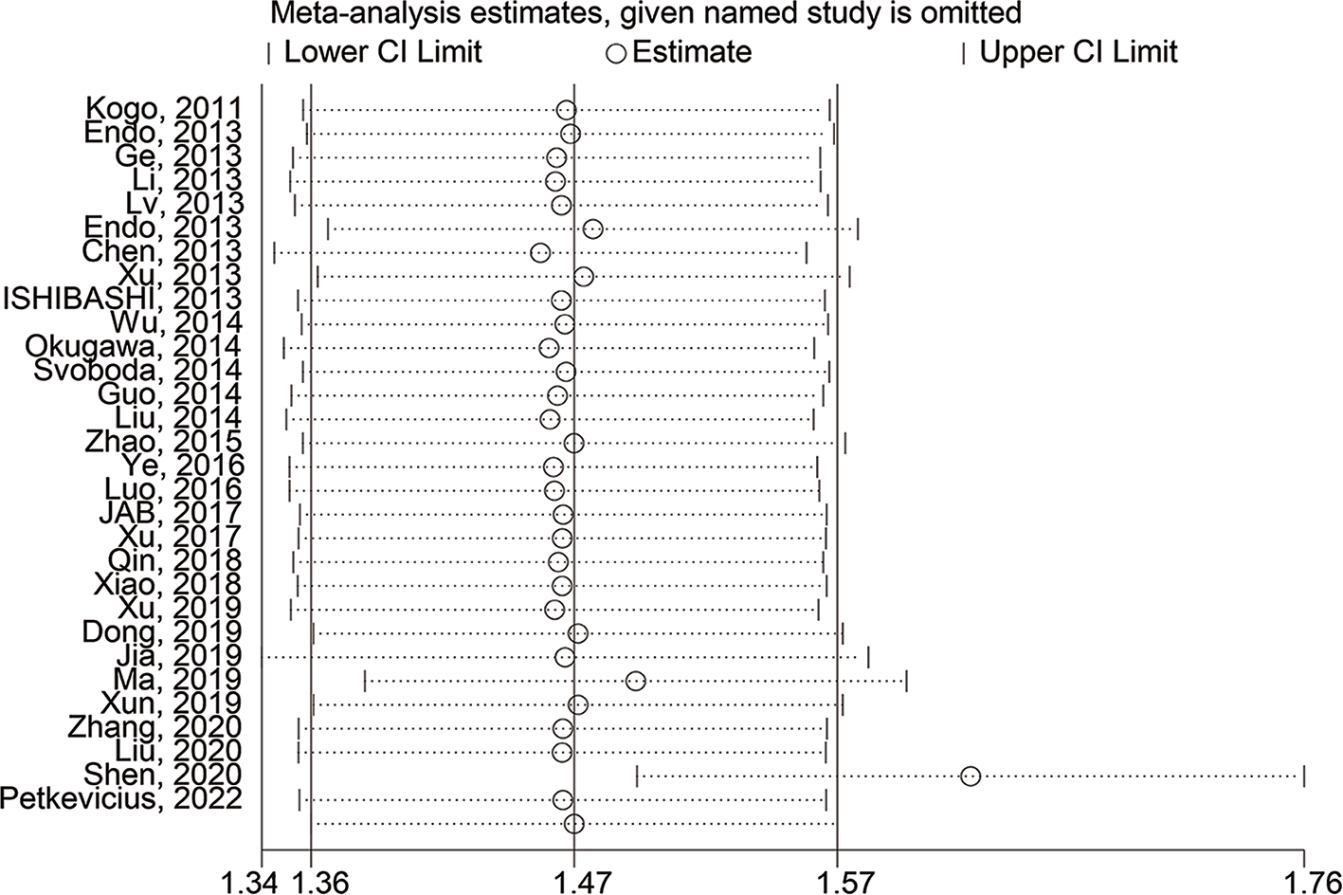
Sensitivity analysis of the meta-analysis The solid line represents the meta-analysis estimates, in which the 95% CI is shown by the width of the dotted horizontal line. Each circle represents a study.

**Figure 4:**
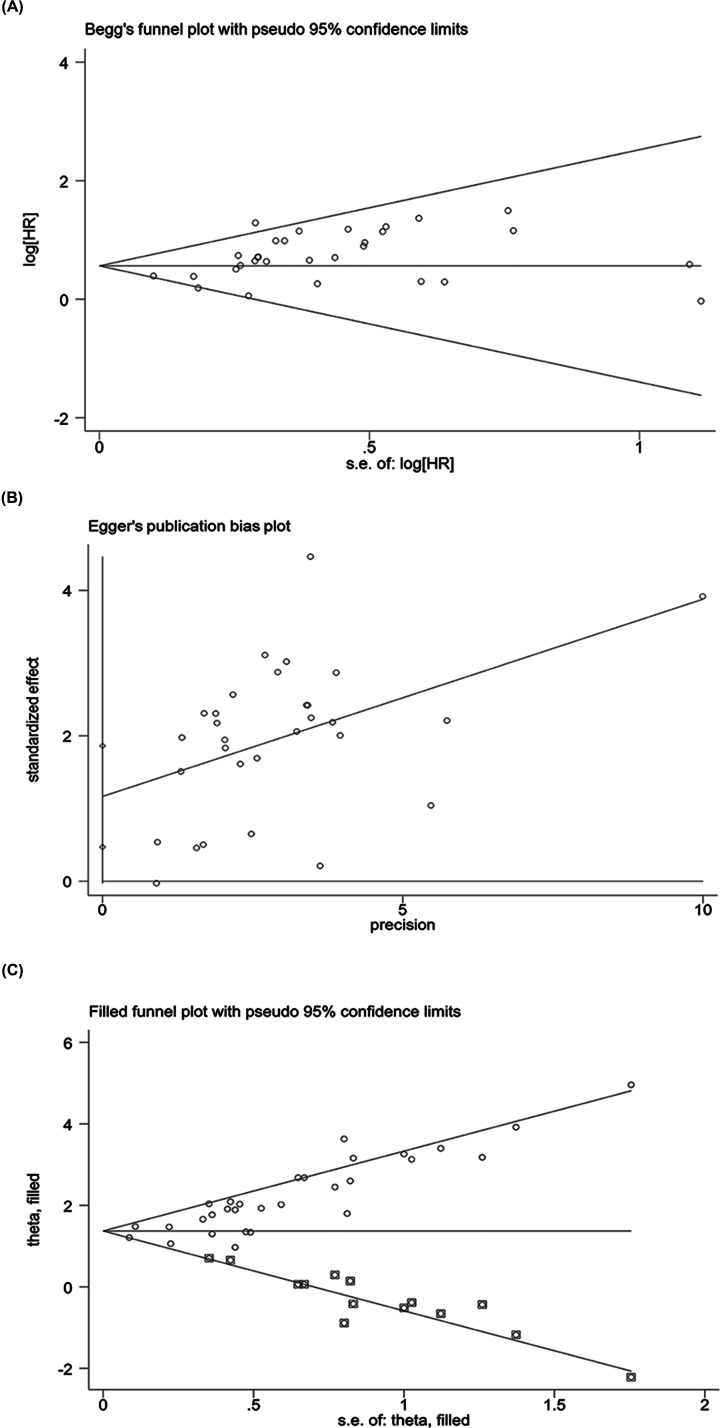
Funnel plots for identifying publication bias (**A**) Begg’s funnel plot analysis. (**B**) Egger’s funnel plot analysis. Each point represents a separate study. (**C**) Trim-and-fill method for testing publication bias. Abbreviations: HR, hazard ratio; S.E., standard error.

OS rates according to the expression level of HOTAIR were reported for 2621 patients in 29 studies. Heterogeneity analysis revealed no heterogeneity among these studies and the fixed-effects model (*I*^2^ = 0.0%, *P*=0.876) was assessed. The results showed that the high HOTAIR expression group had worse outcomes with an HR of 1.56 (95% CI = 1.38, 1.75, *P*<0.001) (Figure 2). To further eliminate heterogeneity, a subgroup meta-analysis was performed, as shown in Table 2. A significant association between HOTAIR and OS was found in China and Japan, with HR values of 1.534 (95% CI = 1.348, 1.720, *P*<0.001) and 2.153 (95% CI = 1.163, 3.143, *P*<0.001), respectively. The association of HOTAIR and OS was significant with sample sizes ≥100 and <100, with HR = 1.580 (95% CI = 1.344, 1.815, *P*<0.001) and HR = 1.539 (95% CI = 1.251, 1.827, *P*<0.001), respectively. In studies that used multivariate analysis, the association was also significant [HR = 1.555 (95% CI = 1.354–1.757, *P*<0.001) or HR = 1.600 (95% CI = 1.171, 2.030, *P*<0.001)], respectively. Furthermore, sensitivity and publication bias analyses were performed using the Begg’s test and Egger’s test. Publication bias was not identified by the Begg’s test (*z* = 1.64, *P*=0.101), but identified the Egger’s test (*t* = 3.43, *P*=0.002). Then, we used the trim and filling method to determine the stability of the analysis results considering the presence of publication bias (Figures 3 and 4). The analysis was considered stable and credible because the results were not reversed before (*P*<0.001) or after (*P*<0.001) trim and filling.

**Table 2:** Pooled HRs for high expression of HOTAIR with respect to OS in different subgroups of patients

Stratified analysis	Studies, *n*	Patients, *n*	Pooled HR (95% CI)	*P*-value	Heterogeneity		
					*I*^2^, %	*P*-value	Model
Tumor type							
Esophageal cancer	6	544	1.94 (1.35, 2.52)	<0.001	0.00	0.856	Fixed effect
Gastric cancer	14	1492	1.58 (1.35, 1.81)	<0.001	0.00	0.781	Fixed effect
Colorectal cancer	7	580	1.46 (0.99, 1.93)	<0.001	0.00	0.683	Fixed effect
Hepatocellular carcinoma	1	64	3.26 (−0.04, 6.56)	0.053	-	-	Fixed effect
Pancreatic cancer	1	78	1.06 (0.45, 1.66)	0.001	-	-	Fixed effect
Cholangiocarcinoma	1	33	1.89 (0.67, 3.11)	0.002	-	-	Fixed effect
Country							
China	22	2119	1.53 (1.35, 1.72)	<0.001	0.00	0.743	Fixed effect
Japan	4	382	2.15 (1.16, 3.14)	<0.001	0.00	0.934	Fixed effect
Denmark	1	40	3.40 (−0.80, 7.60)	0.113	-	-	Fixed effect
Lithuania	1	127	3.13 (−0.69, 6.95)	0.108	-	-	Fixed effect
Czech	1	73	4.46 (−5.68, 15.60)	0.361	-	-	Fixed effect
Sample size							
≥100	10	1187	1.58 (1.34, 1.82)	<0.001	0.00	0.823	Fixed effect
<100	20	1554	1.54 (1.25, 1.83)	<0.001	0.00	0.699	Fixed effect
Analysis type							
Multivariate analysis	17	1907	1.56 (1.35, 1.76)	<0.001	0.00	0.864	Fixed effect
Other	13	834	1.60 (1.17, 2.03)	<0.001	0.00	0.557	Fixed effect

Abbreviations: CI, confidence interval; HR, hazard ratio.

**Figure 5:**
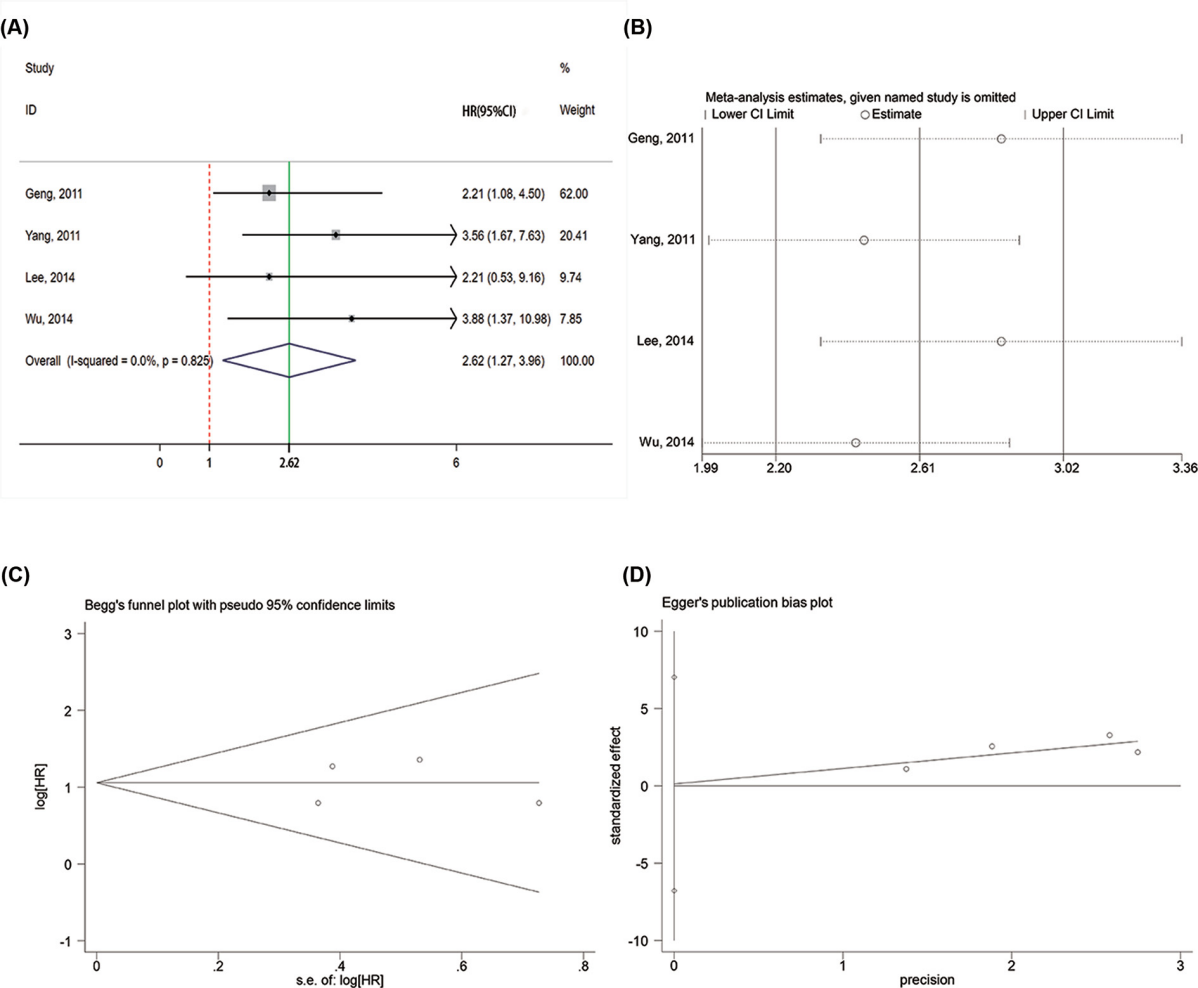
Meta-analysis of pooled HRs to analyze the association between high expression of HOTAIR and DFS (**A**) Forest plot of the studies with DFS data. The solid green line represents the overall summary estimate, in which the 95% CI is given through its width. The dotted vertical red line demonstrates the null value (HR = 1), which represents no increased risk for the outcome. (**B**) Sensitivity analysis. (**C**) Begg’s funnel plot analysis. (**D**) Egger’s funnel plot analysis. Abbreviations: CI, confidence interval; DFS, disease-free survival; HR, hazard ratio; S.E., standard error.

In studies with DFS data, the high HOTAIR group was related to worse outcomes with HR = 2.62 (95% CI = 1.27–3.96, *P*<0.001) and no significant heterogeneity was identified (*I*^2^ = 0%, *P*=0.825) (Figure 5). Subsequently, a sensitivity and publication bias analysis was performed, and the results showed that the analysis was robust and reliable. Subgroup analysis was not performed owing to the limited number of studies.

### Independent prognostic value of HOTAIR for OS in different cancer types

**Figure 6:**
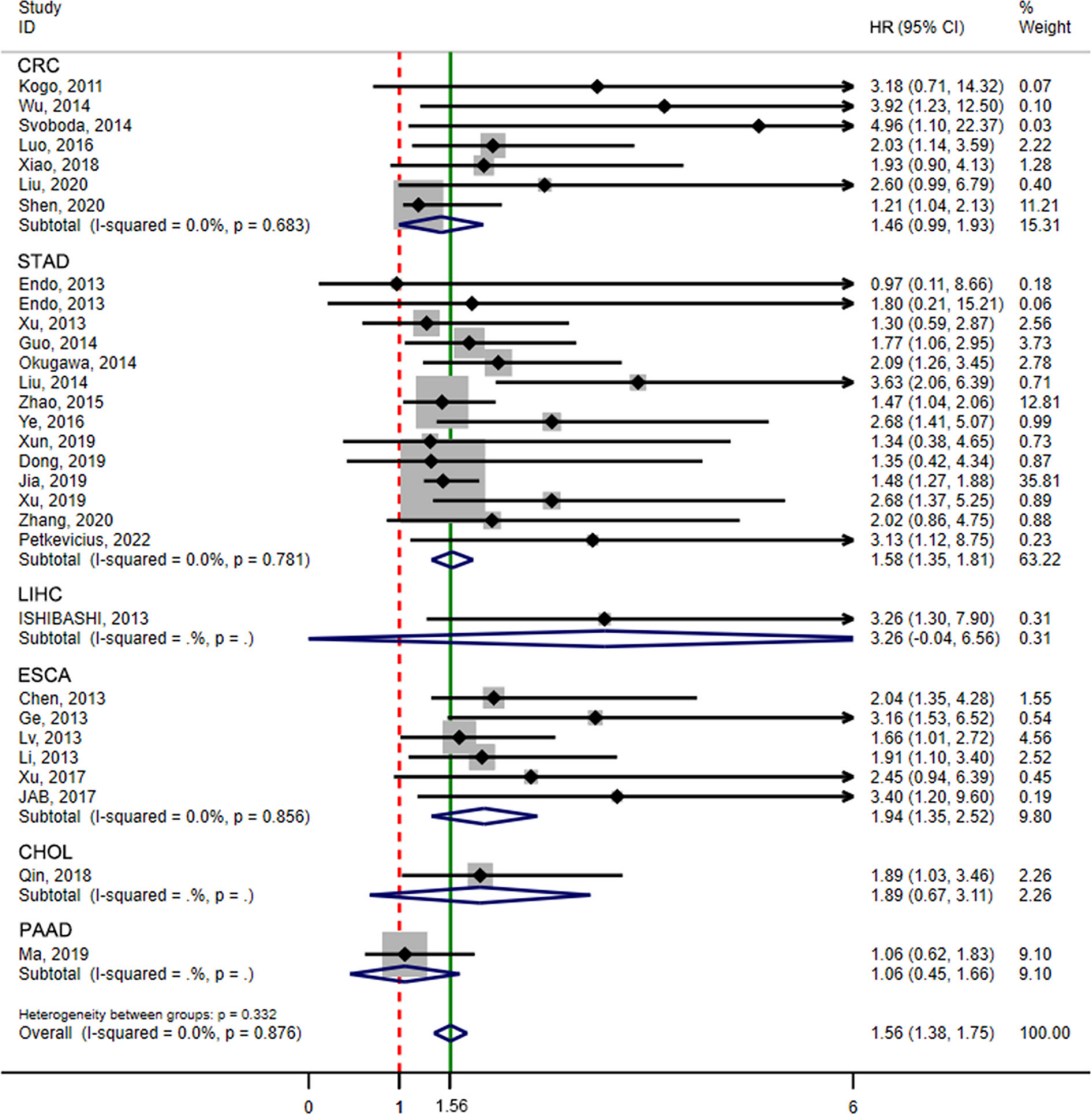
Forest plots to analyze the association between HOTAIR expression and OS in different cancer types The solid green line represents the overall summary estimate, in which the 95% CI is shown by its width. The dotted vertical red line demonstrates the null value (HR = 1), which represents no increased risk for the outcome. Abbreviations: CHOL, cholangiocarcinoma; CI, confidence interval; CRC, colorectal cancer; ESCA, esophageal carcinoma; HR, hazard ratio; LIHC, liver hepatocellular carcinoma; PAAD, pancreatic adenocarcinoma; STAD, stomach adenocarcinoma.

To determine the relationship between the HOTAIR expression level and OS in patients with gastrointestinal system cancer, we classified studies according to the cancer type and analyzed the HR and 95% CI by using the fixed-effects model. The results showed that HOTAIR was a significant prognostic indicator of the OS of patients with ESCA (HR = 1.94, 95% CI = 1.35–2.52, *P*<0.001) and GC (HR = 1.58, 95% CI = 1.35–1.81, *P*<0.001) (Figure 6).

### Construction of lncRNA–mRNA coexpression network to predict potential biological functions of HOTAIR

**Figure 7:**
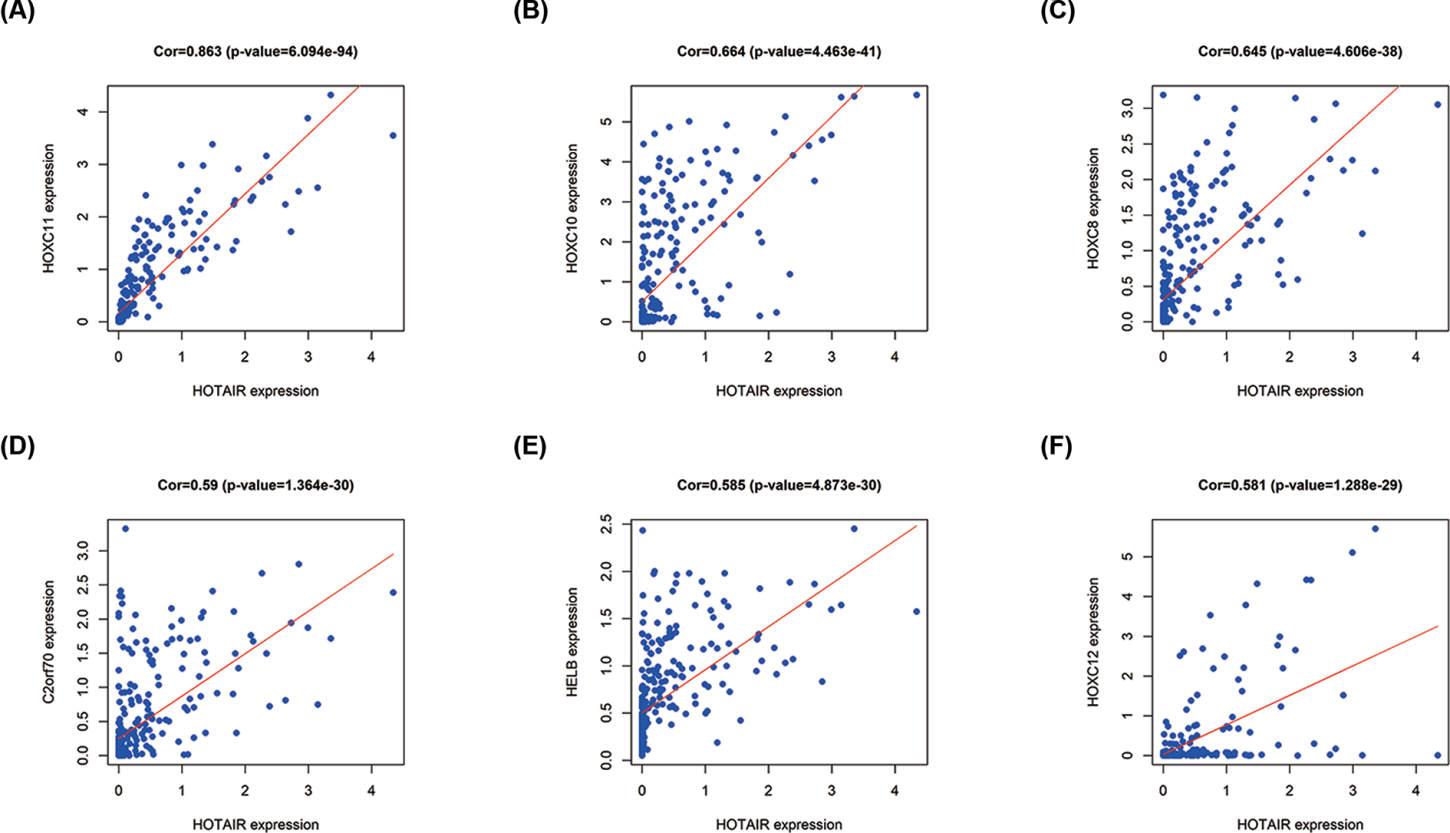
Correlation analysis of PCGs with HOTAIR The top six genes coexpressed with HOTAIR are shown, including *HOXC11* (A), *HOXC10* (B), *HOXC8* (C), *C2orf70* (D), *HELB* (E), and *HOXC12* (F).

**Figure 8:**
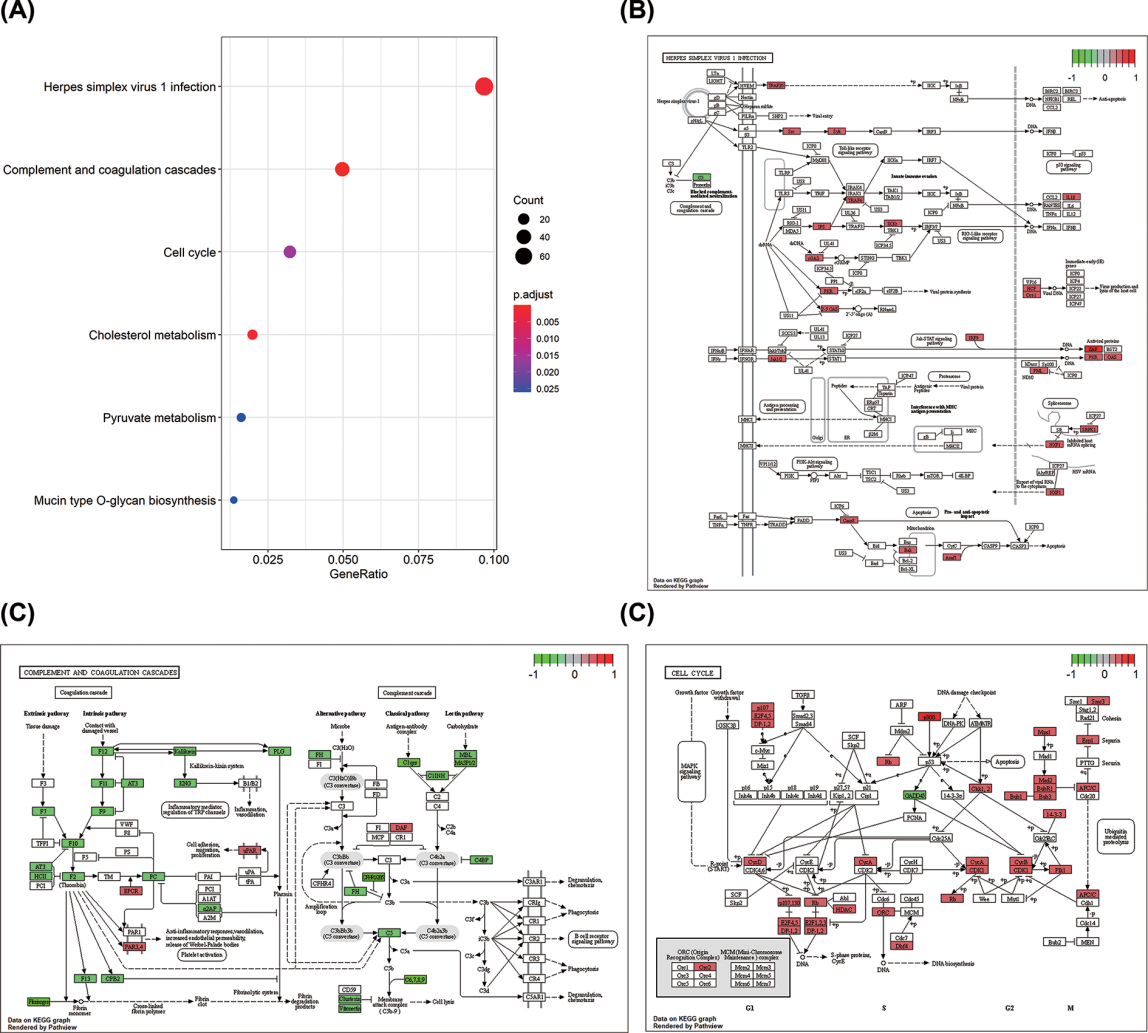
KEGG enrichment analysis of HOTAIR target genes in the KEGG database (**A**) Bubble diagram illustrating the top six KEGG enrichment results, the size of dots indicates the number of target genes, and the different color of dots indicates different *P*-value ranges. (**B**) KEGG pathway enrichment analysis of herpes simplex virus 1 (HSV-1) infection-related gene. HOTAIR mainly activates the Toll-like receptor signaling pathway, the Jak-STAT signaling pathway, and the caspase 8-mediated cell apoptosis pathway. (**C**) KEGG pathway enrichment analysis of complement and coagulation cascades-related genes. HOTAIR impairs the balance of complement activation and promotes inflammation and tumorigenesis resulting in malignant cell proliferation, migration, invasiveness, and metastasis. (**D**) KEGG pathway enrichment analysis of cell cycle-related genes. HOTAIR enhances cyclin D expression, resulting in increased complex formation between cyclin D and CDK4/CDK6, which functions at the G1/S cell cycle transition. The colored bar of the KEGG graphs represents the up-regulation (red) and down-regulation (green) status of each protein.

Using TCGA data, we determined the correlation between the level of expression of lncRNA HOTAIR and each PCG (Figure 7, Supplementary Table S3). Coexpression networks can predict lncRNA function since genes involved in the same biological process are often coexpressed. The results showed that the six PCGs most positively correlated with HOTAIR expression were *HOXC11*, *HOXC10*, *HOXC8*, *C2orf70*, *HELB*, and *HOXC12*, with the correlation coefficient are 0.863, 0.664, 0.645, 0.59, 0.585, and 0.581 (*P*<0.001) (Figure 7). Among them, HOXC11, HOXC10, HOXC8, and HOXC12 interact with HOTAIR in gastrointestinal tumors to jointly promote tumor formation and development [44,83,84]. Next, we investigated the pathways through which HOTAIR significantly accumulates in tumors by mapping genes to KEGG pathways (Figure 8, Supplementary Table S4). HOTAIR is most enriched in the *‘cell cycle’* pathway and pathways relating to infections, namely *‘herpes simplex virus 1 infection’* and *‘complement and coagulation cascades’*, which are associated with key functions of HOTAIR in cancer. The results showed that HOTAIR targets the JAK/STAT signaling pathway, a mitochondria-dependent signaling pathway involving Bcl-2 and Bax, to interfere with tumor growth and apoptosis. Meanwhile, HOTAIR affects coagulation and complement factors, thereby promoting cell migration and proliferation. Additionally, HOTAIR functions in the TGF-β/TAK1/MAPK and P53/P21/Rb signaling pathways and affects the expression of cell cycle-related proteins (CYCA, CYCB, and CYCD), which then affects the cell cycle, leading to faster tumor growth (Figure 8).

### Systematic literature review of the molecular mechanism

**Figure 9:**
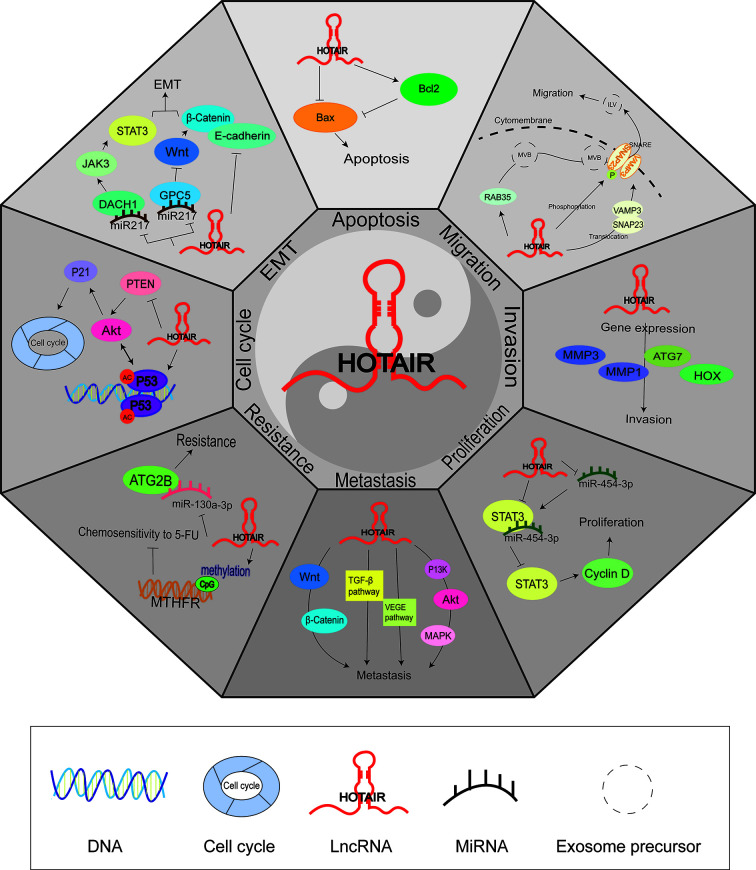
Association between HOTAIR and the development of digestive system malignancies via its regulation ability of cell apoptosis, EMT, cell cycle, drug resistance, metastasis, cell proliferation, invasion, and migration Abbreviations: AKT, serine/threonine kinase 1; ATG2B, autophagy-related 2B; ATG7, autophagy-related 7; Bcl-2, B-cell chronic lymphocytic leukemia/lymphoma-2; Bax, Bcl-2-associated X protein; Cyclin D, D-type cyclins; DACH1, dachshund family transcription factor 1; GPC5, glypican 5; HOXD, homeobox D cluster; JAK3, janus kinase 3; MMP1, matrix metalloproteinase-1; MMP3, matrix metalloproteinase-3; MTHFR, methylenetetrahydrofolate reductase; MVBs, multivesicular bodies; PTEN, phosphatase and tensin homolog; RAB35, member RAS oncogene family; SNAP23, synaptosome associated protein 23; SNARE, soluble N-ethylmaleimide-sensitive fusion factor attachment protein receptor; STAT3, signal transducer and activator of transcription 3; TGF-β, transforming growth factor-β; VAMP3, vesicle-associated membrane protein 3; WNT, wingless-type MMTV integration site family.

The lncRNA HOTAIR plays an important role in the formation and progression of gastrointestinal tumors. Most of the reported effects of HOTAIR on tumors *in vitro* and *in vivo* are related to cell apoptosis, EMT, cell cycle, drug resistance, metastasis, cell proliferation, invasion, and migration (Figure 9). First, studies on gastrointestinal tumors suggest that HOTAIR is vital for cancer cell survival and deficiency of HOTAIR leads to cancer cell apoptosis [69,85]. Meanwhile, HOTAIR can inhibit tumor cell apoptosis via the Bax/Bcl-2 pathway by promoting the expression of related protein Bcl-2 and inhibiting the expression of proto-oncogene Bax [86]. These results are similar to the results of the bioinformatics analysis based on TCGA data in the present study. Second, HOTAIR down-regulates the expression of E-cadherin and miR-217, then activates the DACH1/JAK3/STAT3 and Wnt/β-catenin pathways, which are involved in the tumor’s EMT ability, which is important for primary tumor formation and metastasis [45,63,87]. Third, HOTAIR knockdown can prevent GC cell proliferation, influence cell cycle distribution, and increase P21 and P53 protein levels [70]. HOTAIR also affects the cell cycle by increasing the expression of the P53 tumor-suppressor gene and decreasing the expression of the PTEN tumor-suppressor gene, thereby activating the AKT/P21 signaling pathway and hyperactivating cell division [42]. The role of HOTAIR in influencing the cell cycle was also examined by our bioinformatics analysis. HOTAIR can up-regulate P53, CYCA, and CYCD by increasing the expression of P300. Fourth, many studies have reported a relationship between tumor resistance and HOTAIR. HOTAIR down-regulates Mir-130a-3p and reduces its binding to its target gene ATG2B, thereby affecting tumor chemosensitivity [88]. Studies have also shown that the methylation level of the methylenetetrahydrofolate reductase (MTHFR) gene is regulated by HOTAIR, which reduces the chemosensitivity of cells to 5-fluorouracil [89]. Fifth, HOTAIR also affects tumor metastasis by affecting the Wnt/β-catenin, TGF-β, VAGE, and PI3K/AKT/MAPK pathways; the role and importance of these signaling pathways in the metastatic potential of various cancers have been demonstrated [90,91]. Sixth, HOTAIR promotes cell proliferation by decreasing the expression of miR-454-3p and activating STAT3 and its downstream target gene CYCD [92]. Seventh, HOTAIR participates in regulating the expression of many invasion-related genes, including MMP1 and MMP3 [69]. Eighth, HOTAIR affects intracellular formation and transport of tumor-related exosomes via up-regulation of ras-related protein Rab-35 (RAB35), promotes the phosphorylation of synaptosome-associated protein 23 (SNAP23), and induces the translocation of vesicle-associated membrane protein 3 (VAMP3) and SNAP23 to the cytomembrane, and the formation of transmembrane protein soluble *N*-ethylmaleimide-sensitive fusion factor attachment protein receptor (SNARE), which plays an important role in enhancing the transport of multivesicular bodies (MVBs) related to the formation of exosomes [93]. Overall, HOTAIR participates in gastrointestinal tumor progression through the above eight aspects and may be a potential prognostic factor for digestive system malignancies.

## Discussion

Owing to their potential roles in cancer biology, lncRNAs represent a promising new class for the diagnosis and prognosis of various cancers and have the potential for use as predictors, therapeutic targets, and cancer biomarkers [94]. The different sensitivities of human tumors may be related to different lncRNA expression levels or lncRNA polymorphisms [95]. For example, PCA3 (prostate cancer antigen 3, lncRNA) is considered a biomarker in prostate cancer [96], and the high expression of lncRNAs CCAT2, MALAT1, and NEAT1 is related to worse OS in breast cancer [97]. Moreover, HOTAIR affects the occurrence and development of cancer in many types of malignancies, including digestive system malignant tumors [91]. Though many studies have reported that HOTAIR plays an important role in digestive system tumors [34], the literature lacks an updated systematic analysis and summary. In the present article, we collected studies on HOTAIR in almost all kinds of digestive system malignant tumors and used meta-analysis to clarify the prognostic value of HOTAIR. Furthermore, we used two-sided Pearson correlation coefficients to identify lncRNA HOTAIR-related PCGs. Finally, we predicted and summarized the main related molecular mechanism of the function of HOTAIR in cancer biology by bioinformatics analysis and systematic review.

A meta-analysis of the prognostic value of HOTAIR was reported previously, but the present study only addressed esophageal, gastric, colorectal, and hepatocellular carcinomas [34]. Our review covers the recently published literature on pancreatic cancer and CHOL. The results showed that high HOTAIR expression levels were associated with poorer OS with an HR of 1.56 (95% CI = 1.38–1.75, *P*<0.001). The heterogeneity Q statistic (*P*>0.05) and *I*^2^ value (0.00%) indicate that there is no statistical evidence for heterogeneity among the 32 studies, and the subgroup analysis revealed the relationship between HOTAIR expression and OS, while country, sample size, and analysis methods did not show significant differences. We also performed sensitivity and publication bias analyses. The results of Begg’s and Egger’s tests indicated publication bias between these studies, so the trim and filling method was used to determine the stability of the analysis results. Finally, we classified tumor types and found that high HOTAIR expression was associated with worse OS in STAD and ESCA. HOTAIR may play an important role in these two gastrointestinal tumors. Indeed, a previous meta-analysis demonstrated that patients with up-regulated levels of HOTAIR showed a poor OS rate as compared with patients with lower levels of HOTAIR, suggesting the predictive value of HOTAIR for CRC prognosis [36]. In the present study, the result showed weak significance in CRC as HR is 1.46 (95% CI = 0.99–1.93). Meanwhile, another meta-analysis of HOTAIR in LIHC provides a strict and classic protocol for analysis, although it did not provide specific and detailed analysis data [35]. Due to few studies about HOTAIR in LIHC, our result is also inadequate. Therefore, through our meta-analysis results, we can see that HOTAIR is an important risk factor in well-studied GC, CRC, and ESCA, and can be used as a potential prognostic biomarker. However, there is not enough evidence in tumors such as LIHC, CHOL, and PAAD, and further research is needed.

However, we also noticed that HOTAIR plays an important role in other tumors, not just gastrointestinal tumors [98]. High expression levels of HOTAIR in many cancers, such as lung cancer [25], cervical cancer [26], breast cancer [27], gliomas [28], prostate cancer [99], ovarian cancer [100], and oral cancer [101], have been reported in many studies. In lung cancer, HOTAIR is overexpressed and correlated with tumor metastasis and poor prognosis, which promotes proliferation, survival, invasion, metastasis, and drug resistance in lung cancer cells [25]. In cervical cancer, HOTAIR plays an oncogenic role by promoting cell proliferation, migration, invasion, and autophagy, inhibiting cell apoptosis, stimulating angiogenesis, accelerating cell cycle progression, and inducing EMT [26]. In breast cancer, HOTAIR expression is augmented in primary breast tumors and metastases, and the HOTAIR expression level in primary tumors is a powerful predictor of metastases and death [24]. Therefore, HOTAIR may be a potential therapeutic target in breast cancer [102]. In gliomas, HOTAIR mainly promotes cell proliferation and migration, and inhibits apoptosis. *In vitro* and *in vivo* studies have shown that HOTAIR regulates cell cycle-related genes and related signaling pathways, such as the Wnt/β-catenin axis. Additionally, it can promote angiogenesis and affect the permeability of the blood–brain barrier, thereby modulating the effectiveness of chemotherapeutic drugs [28]. In prostate cancer, a study demonstrated that HOTAIR promotes the invasion and metastasis of PCa by decreasing the inhibitory effect of hepaCAM on MAPK signaling [99]. In ovarian cancer, inhibiting HOTAIR expression in ovarian cancer cells prevents tumorigenesis and metastasis [103], and HOTAIR up-regulates c-Myc in breast and ovarian cancers, thereby promoting cancer cell proliferation [8]. Additionally, HOTAIR is associated with drug susceptibilities, such as platinum resistance [104] and carboplatin resistance [105]. In oral cancer, HOTAIR mediates the suppression of cell proliferation and promotion of apoptosis [106] and promotes the invasion and metastasis of oral squamous cell carcinoma through metastasis-associated gene 2 (MTA2) [107]. Meanwhile, HOTAIR has been extensively studied as an oncogene, and functional SNPs in HOTAIR are associated with cancer risk, including lung, gastric, esophageal, cervical, breast, and prostate cancers [108–111]. Many studies reported that HOTAIR polymorphisms could affect cancer biology. The HOTAIR rs920778 polymorphism is associated with ovarian cancer susceptibility and poor prognosis in the Chinese population [112]. The HOTAIR rs7958904 polymorphism is associated with CRC morbidity and mortality and is a potential CRC biomarker [113]. In conclusion, there is increasing evidence that HOTAIR acts as an oncogene in various cancers and that its up-regulation can lead to malignant transformation and tumorigenesis [67].

Through systematic review and bioinformatics analysis, we found that HOTAIR was involved in the regulation of cancer cell apoptosis, cell proliferation, and the induction of cell cycle arrest, EMT, migration, invasion, metastasis, and resistance in gastrointestinal malignancies. Down-regulation of HOTAIR can induce apoptosis of GC cells and significantly inhibit the proliferation, invasion, and metastasis of GC cells [70], the same findings have been found in other gastrointestinal tumors, although the signaling pathways that may be involved are inconsistent. Meanwhile, we integrated the gene matrix of six major types of Asian patients with gastrointestinal tumors in the TCGA database and found HOTAIR was closely related to the following pathways through the lncRNA–mRNA coexpression network. The *‘cell cycle’* pathway and pathways relating to infections, namely *‘herpes simplex virus-1 infection’* and *‘complement and coagulation cascades’* were significantly enriched in KEGG analysis. Several studies have shown that HOTAIR inhibition leads to G0/G1 cell cycle arrest, thereby inhibiting tumor cell proliferation. Since most GCs are associated with multiple pathogenic infections, several oncogenic viruses play important roles in the malignant progression of GC, and oncolytic viruses appear to be a new therapeutic agent class that inducing antitumor immune responses by selectively killing tumor cells and inducing systemic antitumor immunity [114]. Herpes simplex virus-1 (HSV-1) is a double-stranded DNA virus belonging to the alpha-herpesviruses subfamily, several investigators have used the attenuated HSV vector betaH1 in transplanted human lung, breast, gastric, and colon tumors. Tumor regression and similar apoptosis were observed in all tumors [115]. Finally, coagulation-related genes play an important role in gastrointestinal tumors, and coagulation-related gene models provide new insights and targets for the diagnosis, prognosis prediction, and therapeutic management of patients with GC [116], similar results were also found in CRC [117] and ESCA [118]. In conclusion, our study suggests that HOTAIR has an important association with these pathways and needs to be further elucidated.

## Limitations

Several limitations are unavoidable when performing a meta-analysis. First, publication bias exists due to the more frequent publication of positive results. Second, the results of some small studies are unreliable. Third, different analysis methods were used in the selected studies, and some did not use multivariate analysis. Fourth, the treatment after surgery varied across the selected studies, which might affect the survival time of patients. The relationship between HOTAIR and the survival rate of patients still needs confirmation. Fifth, many studies were performed in Asian populations, especially in China populations, while fewer studies were performed in Europe and America.

## Conclusion

In conclusion, we demonstrated the predictive power of HOTAIR lncRNAs in gastrointestinal malignancies and showed that high HOTAIR expression is associated with worse OS. Then, the molecular mechanisms were systematically evaluated. HOTAIR plays an important role in cancer biology and deserves further study.

## Supplementary Material

Supplementary Tables S1-S5

## Data Availability

The datasets analyzed in the present study are available from the published papers that have been cited in the present manuscript.

## Abbreviations

95% CI: 95% confidence interval
CHOL: cholangiocarcinoma
COAD: colon adenocarcinoma
CRC: colorectal cancer
DFS: disease-free survival
EMT: epithelial-mesenchymal transition
ESCA: esophageal carcinoma
GC: gastric cancer
HOTAIR: HOX transcript antisense intergenic RNA
HOXC: homeobox C cluster
HR: hazard ratio
KEGG: Kyoto Encyclopedia of Genes and Genomes
LIHC: liver hepatocellular carcinoma
LncRNA: long noncoding RNA
NOS: Newcastle–Ottawa quality assessment scale
OS: overall survival
PAAD: pancreatic adenocarcinoma
PCG: protein-coding gene
READ: rectum adenocarcinoma
SNP: single nucleotide polymorphism
STAD: stomach adenocarcinoma
TCGA: The Cancer Genome Atlas
