# Supplementary material for: HOTAIR as a diagnostic and prognostic biomarker of gastrointestinal cancers: an updated meta-analysis and bioinformatics analysis based on TCGA data
Source: Biosci Rep. 2023 Mar 29;43(3):BSR20222174. doi: 10.1042/BSR20222174 (PMC10064413; doi:10.1042/BSR20222174)
Supplement: Supplementary Tables S1-S5 [file BSR-2022-2174_supp.zip › BSR-2022-2174_supps1.docx]

| Database | Search strategy for Pubmed | Search strategy for Embase | Search strategy for Web of science | Search strategy for Scopus | Search strategy for CNKI |
| --- | --- | --- | --- | --- | --- |
| Search strategies | (HOTAIR OR HOX transcript antisense RNA) AND (gastrointestinal cancer OR neoplasm OR cancer OR tumor OR colorectal cancer OR esophageal carcinoma OR pancreatic cancer OR hepatocellular carcinoma OR gastric cancer OR cholangiocarcinoma OR survival OR follow-up OR outcome OR predictor) | ('hotair'/exp OR hotair OR 'hox transcript antisense rna'/exp OR 'hox transcript antisense rna' OR (hox AND transcript AND antisense AND ('rna'/exp OR rna))) AND ('neoplasm'/exp OR neoplasm OR 'cancer'/exp OR cancer OR 'tumor'/exp OR tumor OR 'colorectal cancer'/exp OR 'colorectal cancer' OR (colorectal AND ('cancer'/exp OR cancer)) OR 'esophageal carcinoma'/exp OR 'esophageal carcinoma' OR (esophageal AND ('carcinoma'/exp OR carcinoma)) OR 'pancreatic cancer'/exp OR 'pancreatic cancer' OR (pancreatic AND ('cancer'/exp OR cancer)) OR 'hepatocellular carcinoma'/exp OR 'hepatocellular carcinoma' OR (hepatocellular AND ('carcinoma'/exp OR carcinoma)) OR 'gastric cancer'/exp OR 'gastric cancer' OR (gastric AND ('cancer'/exp OR cancer)) OR 'cholangiocarcinoma'/exp OR cholangiocarcinoma OR 'survival'/exp OR survival OR 'follow up'/exp OR 'follow up' OR 'outcome'/exp OR outcome OR predictor) | ((TS=(HOTAIR OR HOX transcript antisense RNA)) AND TS=(gastrointestinal cancer OR neoplasm OR cancer OR tumor OR colorectal cancer OR esophageal carcinoma OR pancreatic cancer OR hepatocellular carcinoma OR gastric cancer OR cholangiocarcinoma OR survival OR follow-up OR outcome OR predictor)) OR ((AB=(HOTAIR OR HOX transcript antisense RNA)) AND AB=(gastrointestinal cancer OR neoplasm OR cancer OR tumor OR colorectal cancer OR esophageal carcinoma OR pancreatic cancer OR hepatocellular carcinoma OR gastric cancer OR cholangiocarcinoma OR survival OR follow-up OR outcome OR predictor)) | TITLE-ABS-KEY ("HOTAIR" OR "HOX transcript antisense RNA") AND TITLE-ABS-KEY ("gastrointestinal cancer" OR "neoplasm" OR "cancer" OR "tumor" OR "colorectal cancer" OR "esophageal carcinoma" OR "pancreatic cancer" OR "hepatocellular carcinoma" OR "gastric cancer" OR "cholangiocarcinoma" OR "survival" OR "follow-up" OR "outcome" OR "predictor") | (SU%='HOTAIR' + 'HOX transcript antisense RNA') AND (SU%='gastrointestinal cancer' + 'neoplasm' + 'cancer' + 'tumor' + 'colorectal cancer' + 'esophageal carcinoma' + 'pancreatic cancer' + 'hepatocellular carcinoma' + 'gastric cancer' + 'cholangiocarcinoma' + 'survival' + 'follow-up' + 'outcome' + 'predictor') |

**Supplementary Table S1. Search strategies.**
