# Supplementary material for: HOTAIR as a diagnostic and prognostic biomarker of gastrointestinal cancers: an updated meta-analysis and bioinformatics analysis based on TCGA data
Source: Biosci Rep. 2023 Mar 29;43(3):BSR20222174. doi: 10.1042/BSR20222174 (PMC10064413; doi:10.1042/BSR20222174)
Supplement: Supplementary Tables S1-S5 [file BSR-2022-2174_supp.zip › BSR-2022-2174_supps2.docx]

**Supplementary Table S2. Assessment of study quality by NOS.**

| **Author, Year** | **Selection** | | | | **Comparability** | | **Exposure** | | |
| --- | --- | --- | --- | --- | --- | --- | --- | --- | --- |
| Endo, 2013 | 1 | 1 | 1 | 1 | 0 | 1 | 1 | 1 | 0 |
| Guo, 2014 | 1 | 1 | 1 | 1 | 1 | 1 | 1 | 1 | 0 |
| Okugawa, 2014 | 1 | 1 | 1 | 1 | 1 | 1 | 1 | 1 | 0 |
| Xun, 2019 | 1 | 1 | 1 | 1 | 0 | 1 | 1 | 1 | 0 |
| Dong, 2019 | 1 | 1 | 1 | 1 | 0 | 1 | 1 | 1 | 0 |
| Jia, 2019 | 1 | 1 | 1 | 1 | 0 | 1 | 1 | 1 | 0 |
| Lee, 2014 | 1 | 1 | 1 | 1 | 0 | 1 | 1 | 1 | 0 |
| Liu, 2014 | 1 | 1 | 1 | 1 | 0 | 1 | 1 | 1 | 0 |
| Zhang, 2020 | 1 | 1 | 0 | 1 | 0 | 1 | 1 | 1 | 0 |
| Petkevicius, 2022 | 1 | 1 | 0 | 1 | 0 | 1 | 1 | 1 | 0 |
| Xu, 2013 | 1 | 1 | 1 | 1 | 0 | 1 | 1 | 1 | 0 |
| Xu, 2019 | 1 | 1 | 1 | 1 | 0 | 1 | 1 | 1 | 0 |
| Ye, 2016 | 1 | 1 | 1 | 1 | 0 | 1 | 1 | 1 | 0 |
| Zhao, 2015 | 1 | 1 | 1 | 1 | 0 | 1 | 1 | 1 | 0 |
| Ma, 2019 | 1 | 1 | 0 | 1 | 1 | 1 | 1 | 1 | 0 |
| Ishibashi, 2013 | 1 | 0 | 1 | 1 | 0 | 1 | 1 | 1 | 0 |
| Geng, 2011 | 1 | 1 | 1 | 1 | 0 | 1 | 1 | 1 | 0 |
| Yang, 2011 | 1 | 1 | 1 | 1 | 0 | 1 | 1 | 1 | 0 |
| Chen, 2013 | 1 | 1 | 1 | 1 | 0 | 1 | 1 | 1 | 0 |
| Ge, 2013 | 1 | 1 | 1 | 1 | 0 | 1 | 1 | 1 | 0 |
| Xu, 2017 | 1 | 0 | 1 | 1 | 0 | 1 | 1 | 1 | 0 |
| Qin, 2018 | 1 | 1 | 1 | 1 | 0 | 1 | 1 | 1 | 0 |
| Jab, 2017 | 1 | 1 | 1 | 1 | 0 | 1 | 1 | 1 | 0 |
| Lv, 2013 | 1 | 1 | 0 | 1 | 1 | 0 | 1 | 1 | 0 |
| Li, 2013 | 1 | 0 | 0 | 1 | 0 | 1 | 1 | 1 | 0 |
| Kogo, 2011 | 1 | 1 | 1 | 1 | 1 | 0 | 1 | 1 | 0 |
| Liu, 2020 | 1 | 1 | 1 | 1 | 0 | 1 | 1 | 1 | 0 |
| Luo, 2016 | 1 | 1 | 1 | 1 | 0 | 1 | 1 | 1 | 0 |
| Wu, 2014 | 1 | 1 | 1 | 1 | 0 | 1 | 1 | 1 | 0 |
| Jia, 2020 | 1 | 1 | 1 | 1 | 0 | 1 | 1 | 1 | 0 |
| Xiao, 2018 | 1 | 1 | 1 | 1 | 0 | 1 | 1 | 1 | 0 |
| Svoboda, 2014 | 1 | 1 | 1 | 1 | 1 | 1 | 1 | 1 | 0 |
